# Altered Lipid Metabolism in the Vaginal Wall of Women With Pelvic Organ Prolapse: A Targeted Lipidomics Study

**DOI:** 10.1096/fj.202502840R

**Published:** 2025-11-28

**Authors:** Shufei Zhang, Hanyue Li, Xiaoyu Tian, Hongyang Xue, Yong He, Nuo Jiang, Li Hong

**Affiliations:** ^1^ Department of Obstetrics and Gynecology Renmin Hospital of Wuhan University Wuhan Hubei People's Republic of China

**Keywords:** glycerophospholipid, lipid metabolism, pelvic organ prolapse, targeted lipidomics, UHPLC–MS/MS

## Abstract

Pelvic organ prolapse (POP) severely impacts quality of life, but its association with lipid metabolism remains unclear. This study explored lipidomic alterations in vaginal anterior wall tissues of POP patients. We performed UHPLC–MS/MS–based targeted lipidomics on vaginal anterior wall tissues from 8 POP patients and 8 matched controls. Multivariate analyses (PCA, PLS‐DA, OPLS‐DA) identified significantly altered lipids, and KEGG pathway analysis determined related metabolic pathways. Among 1010 identified lipids, POP tissues exhibited significant lipid remodeling: phosphatidic acid (PA) increased from 0.048% to 0.226%, triglycerides (TG) decreased from 94.901% to 83.927%. Forty‐ four lipids were significantly altered (VIP > 1, *p* < 0.05), with KEGG analysis highlighting enrichment in glycerophospholipid metabolism. Reprogramming of lipid chain length and saturation further confirmed metabolic dysregulation. POP may be associated with disrupted lipid metabolism, characterized by elevated PA/PE/PC and reduced TG, potentially impairing membrane stability, mitochondrial function, and energy homeostasis. These findings reveal novel molecular mechanisms in POP and suggest lipid metabolism as a therapeutic target.

## Graphical abstract

This graphical abstract illustrates the association between pelvic organ prolapse (POP) and obesity. Vaginal anterior wall tissues were collected for targeted lipidomics analysis, revealing significant alterations in lipid metabolism and identification of differential lipid species in POP.

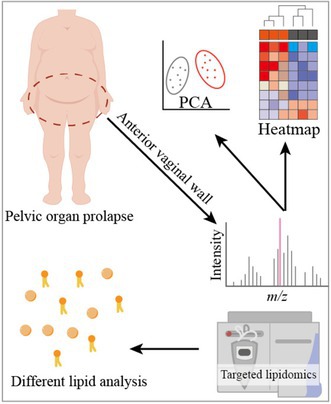

## 1 Introduction

Pelvic floor dysfunction is a group of common conditions caused by damage to the pelvic floor support structures or neuro‐muscular dysfunction. It primarily includes pelvic organ prolapse (POP), stress urinary incontinence, and fecal incontinence [1]. Epidemiological studies show that nearly 25% of women in the United States suffer from PFD, while the prevalence of SUI and symptomatic POP among women in China is as high as 18.9% and 9.6%, respectively [2]. With the accelerated aging of the population and the adjustment of national fertility policies, China has entered a “multichild era,” leading to an increased likelihood of multiple pregnancies and deliveries among women. This makes the study of the pathogenesis of POP, as well as prevention and treatment strategies, of significant clinical and social importance [3, 4].

Studies have demonstrated that pregnancy and childbirth are major risk factors for POP, with aging, obesity, and other factors also playing important roles in the pathogenesis of POP. Studies by Si et al. have found that both central obesity and noncentral overweight are risk factors for POP [5]. Moreover, Mendelian randomization studies have further established a significant causal relationship between body mass index, serum low‐density lipoprotein, triglyceride levels, and POP [6]. Similarly, as a pelvic floor dysfunction disorder, Zhu et al. found that hyperlipidemia is significantly associated with an increased risk of stress urinary incontinence in obese women (OR = 1.52, 95% CI = 1.03–2.25), and that hyperlipidemia and obesity exhibit a significant synergistic effect on stress urinary incontinence. These findings suggest that lipid metabolism may play a critical role in the onset and progression of POP.

In summary, we propose that lipid metabolism may be closely related to the occurrence and progression of POP. However, there is currently no systematic study exploring the specific relationship between various lipids and POP. Therefore, this study employed targeted lipidomic sequencing technology to analyze the anterior vaginal wall tissues of women with POP, aiming to comprehensively identify lipid species and metabolic pathways potentially involved in POP, with the goal of providing new molecular targets and theoretical foundations for the prevention and treatment of POP.

## 2 Materials and Methods

### 2.1 Study Population

A total of 16 female patients were selected from the Obstetrics and Gynecology Department of Renmin Hospital of Wuhan University, China, between January 1, 2025, and February 1, 2025. The cohort consisted of 8 POP patients and 8 non‐POP patients. Patient demographics and clinical characteristics are presented in Table 1. Inclusion criteria: Patients who signed informed consent, women over 50 years of age, and who underwent total hysterectomy; for the POP group, patients with an Aa > +1 on the POP‐Q scale. Exclusion criteria: Patients with severe systemic diseases or other conditions affecting lipid metabolism (such as diabetes, liver disease, etc.), or those using medications that could affect lipid metabolism. This study was approved by the Clinical Research Ethics Committee of Renmin Hospital of Wuhan University (WDRY2025‐K018). All procedures were performed in accordance with the ethical standards of the Declaration of Helsinki. Written informed consent was obtained from all participants and/or their legal guardians before sample collection.

**TABLE 1:** Comparison of characteristics between the two groups.

Variable	Control (*n* = 8)	POP (*n* = 8)	*p*
Age (years)	63.5 ± 3.51	64.25 ± 1.49	0.59
BMI (kg/m^2^)	24.93 ± 1.29	25.28 ± 2.06	0.69
Menopausal status	8 (100%)	8 (100%)	1.0
Hormone replacement therapy	1 (12.5%)	1 (12.5%)	1.0
Vaginal parity	1.88 ± 0.64	2.25 ± 0.71	0.28
Comorbidities	1 (12.5%)	2 (25.0%)	0.52

### 2.2 Sample Processing

Sample Storage: Following the collection of vaginal anterior wall tissue samples, blood and excess connective tissue were promptly removed. The samples were subsequently placed in liquid nitrogen for 15 min and stored at −80°C until all samples were collected and sent to APTBIO Co. Ltd. (Shanghai, China) for targeted lipidomics analysis.

Quality Control (QC) Sample Preparation: Equal volumes from each group were mixed to create a QC sample. The QC sample was used to assess instrument status and equilibrium of the chromatography‐mass spectrometry system before analysis and was also interspersed during the sample analysis process to evaluate system stability throughout the experiment.

Sample Preprocessing: After slowly thawing the samples at 4°C, an appropriate amount was mixed with 200 μL of methanol, vortexed, and then 10 μL of internal standard mixture was added. Next, 800 μL of methyl tert‐butyl ether (MTBE) was added, followed by vortexing and ultrasonic treatment in a low‐temperature water bath for 20 min. The sample was allowed to stand at room temperature for 30 min, then 200 μL of mass spectrometry‐grade water was added, vortexed, and centrifuged at 14000 rpm for 15 min at 4°C. The upper organic phase was collected, evaporated under nitrogen, and redissolved in 200 μL of 90% isopropanol/acetonitrile solution for mass spectrometry analysis. The sample was vortexed again, and centrifuged at 14000 rpm for 15 min at 4°C, after which the supernatant was collected for analysis.

### 2.3 Chromatography‐Mass Spectrometry Analysis

Chromatographic Conditions: The analysis was conducted using an LC‐30 ultra‐high‐performance liquid chromatography (UHPLC) system with C18 and Amino columns. For the C18 column, the column temperature was set to 45°C with a flow rate of 0.35 mL/min. The mobile phase consisted of A: 70% acetonitrile +30% water +5 mM ammonium acetate, and B: isopropanol solution. The gradient elution program was as follows: from 0 to 5.0 min, B was increased from 20% to 60%; from 5.0 to 13.0 min, B was increased from 60% to 100%; and from 13.1 to 17.0 min, B was maintained at 20%. For the Amino column, the column temperature was set to 40°C with a flow rate of 0.4 mL/min. The mobile phase consisted of A: 2 mM ammonium acetate +50% methanol +50% acetonitrile, and B: 2 mM ammonium acetate +50% acetonitrile +50% water. The gradient elution program was as follows: from 0 to 3.0 min, B was maintained at 3%; from 3.0 to 13.0 min, B was increased from 3% to 100%; from 13.0 to 17.0 min, B was maintained at 100%; and from 17.1 to 22.0 min, B was maintained at 3%. Samples were kept at 10°C in an automatic sampler throughout the analysis. To reduce variability caused by instrument signal fluctuations, samples were analyzed in random order.

Mass spectrometry analysis was performed using both positive and negative ion modes on an AB 6500+ QTRAP mass spectrometer (AB SCIEX). The electrospray ionization (ESI) source conditions were as follows: source temperature was set to 400°C, Ion Source Gas1 (GS1) was set to 50, Ion Source Gas2 (GS2) was set to 55, Curtain Gas (CUR) was set to 35, and IonSpray Voltage (IS) was +3000 V for positive mode or −2500 V for negative mode. Multiple Reaction Monitoring (MRM) mode was employed for monitoring.

### 2.4 Quality Control

The Sciex OS software was employed to extract peaks from the MRM raw data, obtaining the peak area and the ratio of the internal standard peak area for each substance, and subsequently calculating the concentration of each substance. QC sample evaluation involved several steps. Total Ion Chromatogram (TIC) comparison was performed by overlapping and comparing the TICs from QC samples. Overlapping peak intensities and retention times indicated minimal variations caused by instrument errors. Pearson correlation analysis was conducted on the QC samples, and a correlation coefficient greater than 0.9 was considered indicative of good correlation. Principal Component Analysis (PCA) was applied to the ion peaks extracted from all experimental and QC samples after Pareto scaling. Hotelling's T2 test was used to detect outlier samples, with a 95% or 99% confidence interval. A Multivariate Control Chart (MCC) was constructed using the ion peaks from QC samples to monitor the instrument's stability. Finally, the Relative Standard Deviation (RSD) of the ion peak abundance in QC samples was assessed; smaller RSD values indicated better instrument stability and were considered a key indicator of data quality.

### 2.5 Data Analysis

Data Preprocessing: the data extracted from LipidSearch, lipids with relative standard deviation (RSD) greater than 30% and missing values greater than 50% in each group were removed.

Data Analysis: The analysis included identification statistics, lipid composition analysis, and lipid differential analysis. Lipid composition analysis included the assessment of lipid subclass composition and lipid content distribution. Lipid differential analysis included the examination of lipid content, chain length, chain saturation, and KEGG pathway enrichment. Unless otherwise specified, all the above analyses were performed using R (version 4.2.1).

## 3 Results

### 3.1 Lipid Composition Analysis

**FIGURE 1:**
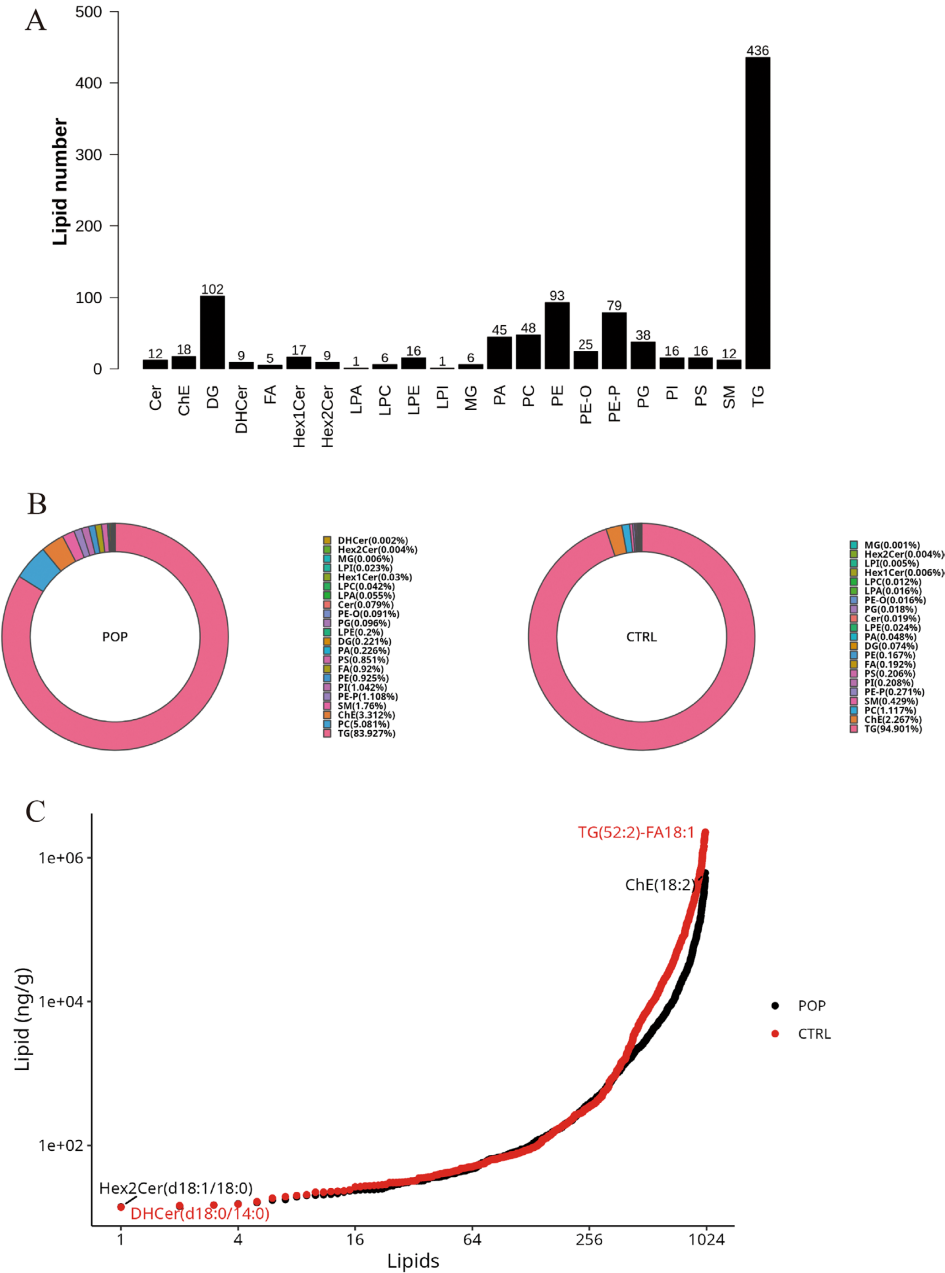
Lipid Composition Analysis. (A) Statistics of lipid subclasses and lipid molecule counts; (B) Composition of lipid subclasses; (C) Dynamic distribution range of lipid content, the highest and lowest abundant lipid subclasses were identified in each group.

In this study, over 2400 lipid species, including glycerophospholipids, sphingolipids, glycerolipids, sterol esters, fatty acyls, and glycolipids, were quantitatively measured. A total of 1010 lipid molecules were detected, with the highest number of detected species being triglycerides (TG), with 436 types identified, followed by diglycerides (DG) and phosphatidylethanolamines (PE) (Figure 1A). First, we compared the total lipid content between the two groups, and the results showed no significant difference between the control group and the POP group in terms of total lipid content (*p* = 0.0765) (Figure S1A). Next, we analyzed the lipid composition of the two groups. The most abundant lipids were TG, plasmalogen phosphatidylethanolamine (PE‐P), phosphatidylcholine (PC), cholesterol esters (ChE), and sphingomyelin (SM). Compared to the control group, the proportion of PC in the POP group significantly increased from 1.117% to 5.081%, while the proportion of TG decreased from 94.901% to 83.927% (Figure 1B). A dynamic analysis of lipid content revealed that the most abundant lipid molecule in the POP group was ChE (18:2), while the least abundant was Hex2Cer (d18:1/18:0). In the control group, the highest lipid molecule content was TG (52:2)‐FA18:1, and the lowest was DHCer (d18:0/14:0) (Figure 1C).

### 3.2 Lipid Subclass Level Differences in POP

**FIGURE 2:**
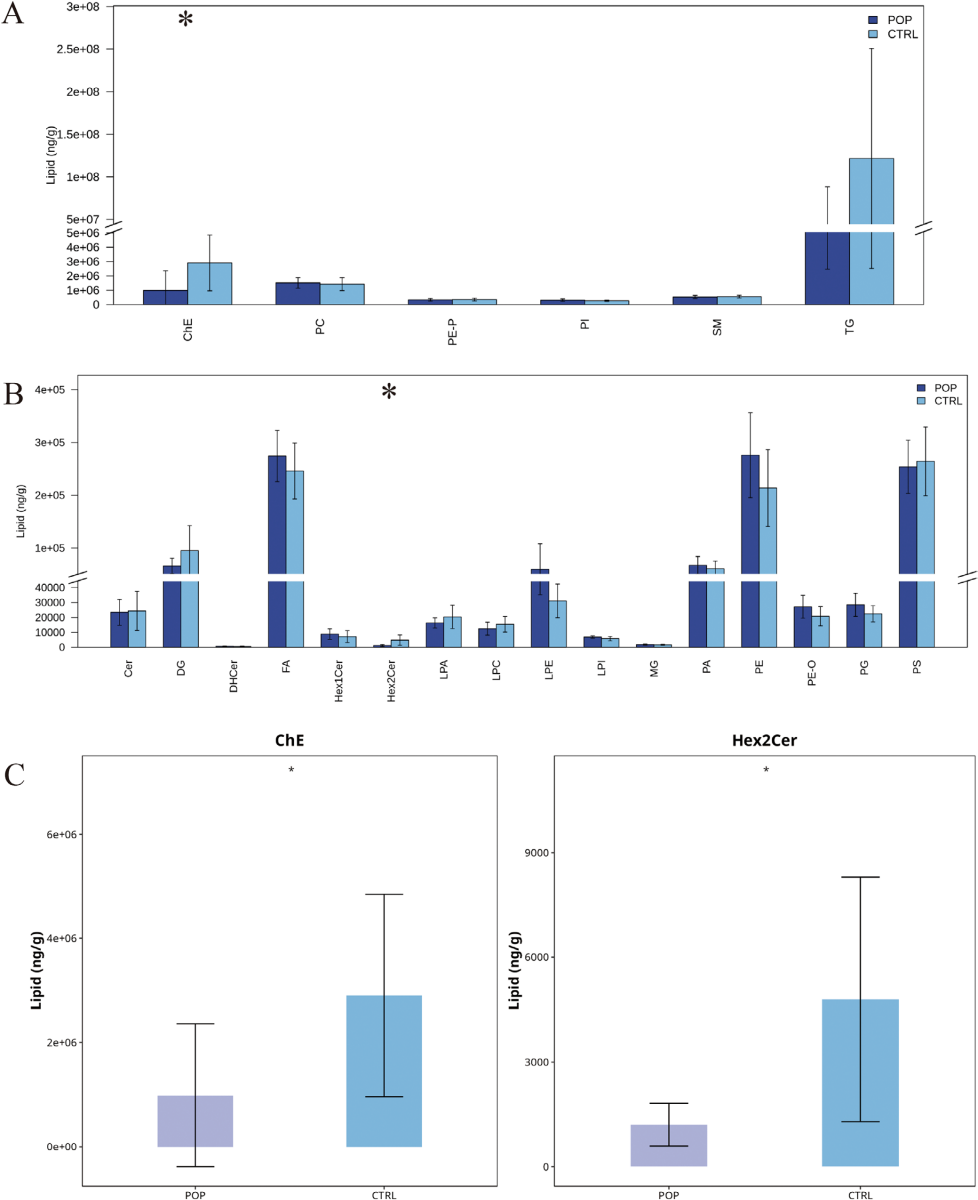
Lipid Subclass Content Analysis. (A) Changes in the content of highly expressed lipid subclasses; (B) Changes in the content of lowly expressed lipid subclasses; (C) Lipid subclasses with significantly different content.

Alterations in lipid subclass content reflect changes in lipid function. Therefore, we compared the expression changes of lipid subclasses in different samples. Results showed that among lipid subclasses with high absolute content, ChE expression was significantly reduced in the POP group. Among lipid subclasses with lower absolute content, Hex2Cer expression was significantly reduced. Most other lipid subclasses, such as TG, PA, and PE, showed no significant content differences (Figure 2A–C).

### 3.3 Univariate Differential Analysis of Lipid Molecules in POP

**FIGURE 3:**
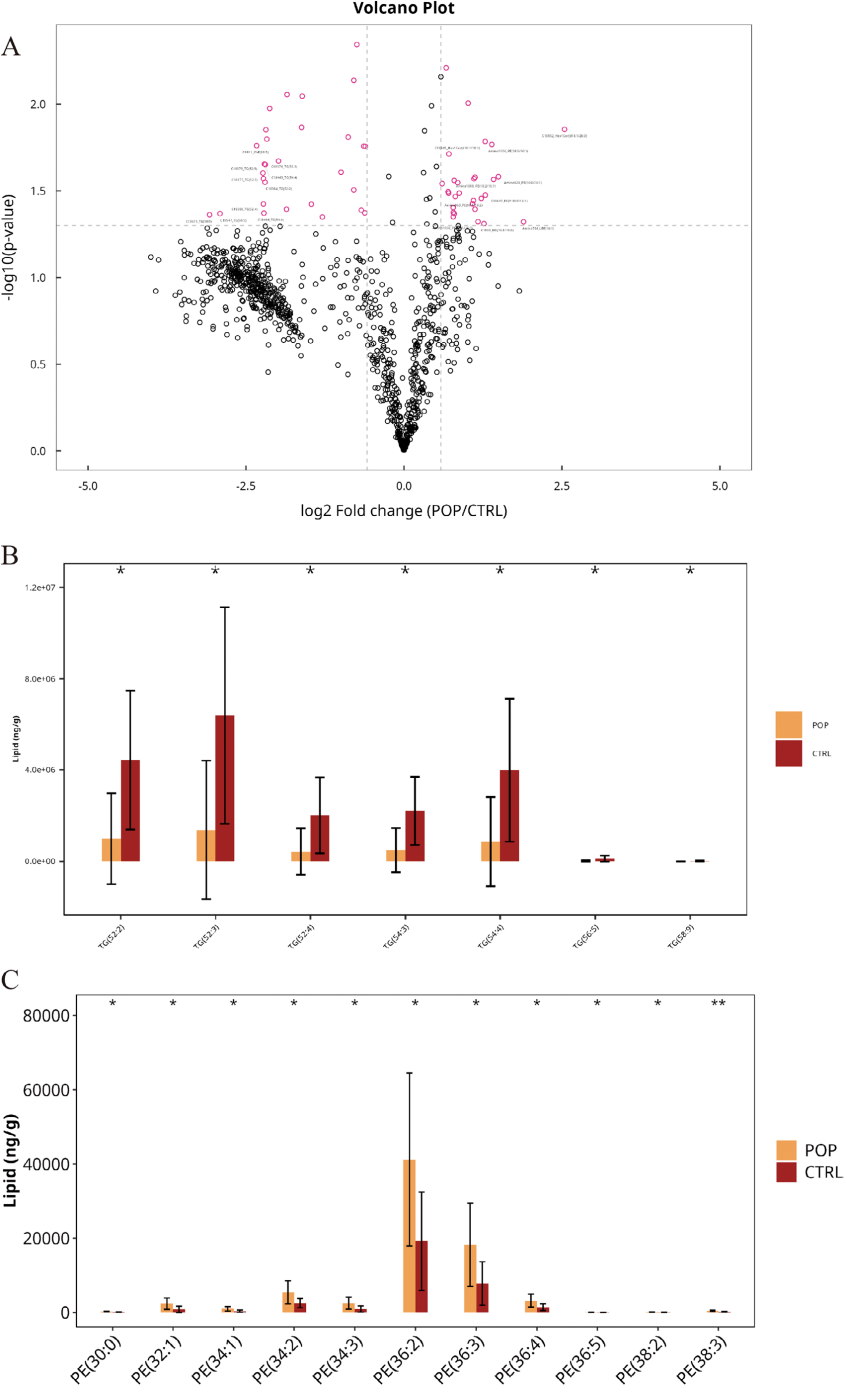
Univariate Statistical Analysis. (A) Univariate analysis was performed on all detected lipid molecules to identify differential lipids, and the results were visualized using a volcano plot; (B) Statistical chart of differential TG molecules; (C) Statistical chart of differential PE molecules.

After comparing lipid subclasses, we performed univariate differential analysis between the two groups. The results identified lipid molecules with FC > 1.5 or FC < 0.67 and *p* value < 0.05, which were marked in pink, with the top 10 most upregulated and downregulated lipid molecules annotated (Figure 3A).

Compared to the control group, the ten most significantly downregulated lipid molecules in the POP group were: TG (58:9)‐FA20:4, TG (56:5)‐FA20:4, ChE (20:5), TG (52:3)‐FA16:0, TG (52:4)‐FA18:2, TG (52:3)‐FA18:2, TG (54:4)‐FA18:2, TG (54:4)‐FA18:1, TG (52:2)‐FA16:0, and TG (52:3)‐FA18:1. The ten most significantly upregulated lipid molecules were: Hex1Cer (d18:1/26:0), LPE (16:1), PE (16:0/16:1), PE (18:2/16:1), PE (18:0/16:1), PE (P‐18:0/16:1), Hex1Cer (d18:1/18:1), DG (16:1/18:0), PE (18:1/18:2), and PC (18:1/16:1) (Table 2).

**TABLE 2:** Univariate statistical analysis of the top 10 most significantly up‐ and down‐regulated lipid molecules.

Univariate statistical analysis	Lipid name	Class	Fold change	*p*
Upward	Hex1Cer (d18:1/26:0)	SP	5.82	0.014
	LPE (16:1)	GP	3.71	0.048
	PE (16:0/16:1)	GP	2.82	0.026
	PE (18:2/16:1)	GP	2.67	0.027
	PE (18:0/16:1)	GP	2.62	0.017
	PE (P‐18:0/16:1)	GP	2.44	0.033
	Hex1Cer (d18:1/18:1)	SP	2.44	0.016
	DG (16:1/18:0)	GL	2.41	0.049
	PE (18:1/18:2)	GP	2.33	0.035
	PC (18:1/16:1)	GP	2.25	0.048
Downward	TG (58:9)‐FA20:4	GL	0.12	0.04
	TG (56:5)‐FA20:4	GL	0.13	0.04
	ChE (20:5)	ST	0.20	0.02
	TG (52:3)‐FA16:0	GL	0.21	0.02
	TG (52:4)‐FA18:2	GL	0.21	0.04
	TG (52:3)‐FA18:2	GL	0.21	0.03
	TG (54:4)‐FA18:2	GL	0.22	0.04
	TG (54:4)‐FA18:1	GL	0.22	0.02
	TG (52:2)‐FA16:0	GL	0.22	0.03
	TG (52:3)‐FA18:1	GL	0.22	0.02

Regarding each subclass, 31 lipid molecules were downregulated in the POP group compared to the control group. Among these, TG had 11 molecules, including TG (52:2)‐FA16:0, TG (52:3)‐FA16:0, TG (52:3)‐FA18:1, and TG (52:3)‐FA18:2; DG had 6 molecules, including DG (16:0/18:3), DG (18:1/20:0), and DG (18:1/20:4); LPC had 1 molecule, LPC (16:0); ChE had 5 molecules, including ChE (20:5), ChE (22:6), and ChE (20:3); Hex1Cer and Hex2Cer had 4 molecules, including Hex1Cer (d18:0/24:1) and Hex2Cer (d18:1/16:0); PE had 3 molecules, PE (P‐20:1/18:1) and PE (P‐20:1/20:3); PG had 1 molecule, PG (20:0/22:5) (Figure 3B and Table S1).

In contrast, 38 lipid molecules were upregulated in the POP group compared to the control group. PC had 3 molecules, including PC (18:1/16:1). PE had 18 molecules, including PE (16:0/14:0), PE (18:0/16:1), PE (18:1/16:1), and PE (18:2/16:1); PA had 2 molecules, PA (16:0/16:1) and PA (18:1/20:1); LPE had 2 molecules, LPE (20:1) and LPE (16:1); PI had 6 molecules, including PI (16:0/18:1), PI (16:0/18:2), and PI (18:0/18:1); Hex1Cer had 3 molecules, including Hex1Cer (d18:1/18:1), Hex1Cer (d18:1/26:0), and Hex1Cer (d18:0/26:0); DG had 2 molecules, DG (16:1/18:0) and DG (18:0/20:2); PG had 1 molecule, PG (22:6/22:6); DHCer had 1 molecule, DHCer (d18:0/14:0) (Figure 3C and Table S1).

### 3.4 Multivariate Statistical Analysis

Principal component analysis (PCA) demonstrated clear separation between the POP and control group samples. The PCA model parameter *R* ^2^ obtained through 7‐fold cross‐validation was 0.782, indicating that the model is stable and reliable (Figure S1B & Table 3). Additionally, partial least squares discriminant analysis (PLS‐DA) was conducted, and the model parameter *Q* ^2^ obtained through 7‐fold cross‐validation was 0.708, suggesting a stable and reliable model. Permutation testing showed that as the permutation retention decreased, the *R* ^2^ and *Q* ^2^ of the random model gradually decreased, indicating that the original model was not overfitted, and its robustness was good (Figure S1C,D and Table 3).

**TABLE 3:** Model evaluation parameters for PCA, PLS‐DA, and OPLS‐DA.

Type	A	*N*	R2X (cum)	R2Y (cum)	Q2 (cum)
PCA	2	16	0.782	—	—
PLS‐DA	3	16	0.803	0.934	0.708
OPLS‐DA	1 + 3	16	0.83	0.979	0.668

**FIGURE 4:**
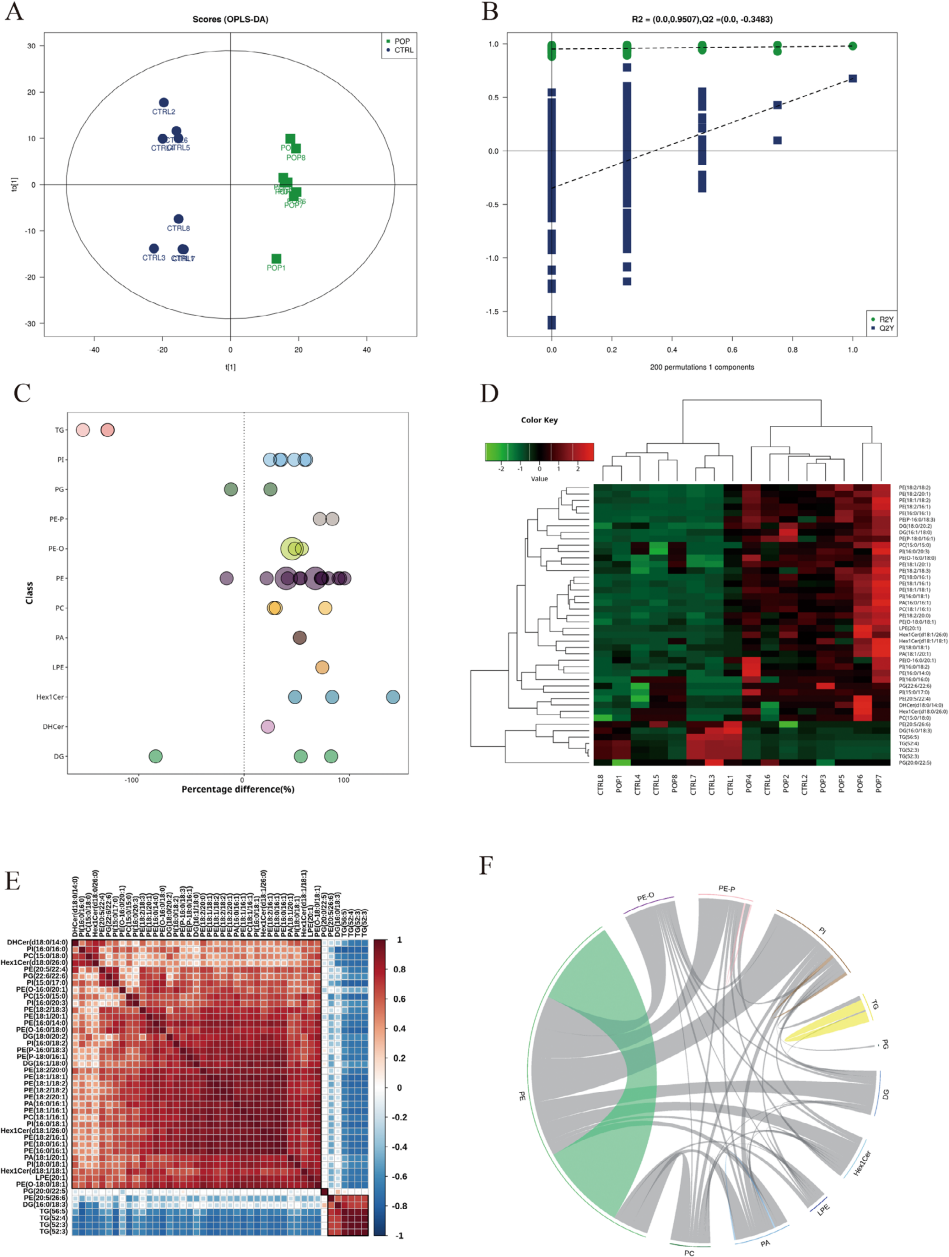
Multivariate Statistical Analysis. (A) OPLS‐DA plot; (B) OPLS‐DA permutation test plot; (C) Bubble plot of significantly different lipid molecules; smaller bubbles represent significant differences (0.01 < *p*‐value < 0.05), larger bubbles represent highly significant differences (*p*‐value < 0.01); (D) Heatmap of significantly different lipid molecules; (E) Correlation heatmap of significantly different lipid molecules, with red indicating positive correlation, blue indicating negative correlation, and the color intensity corresponding to the absolute value of the correlation coefficient (the higher the correlation, the darker the color); (F) Chord diagram of significantly different lipid molecules, where the inner circle links represent the significantly different lipid molecules and the outer arcs represent lipid subclasses, with colored lines indicating correlations within subclasses, and dark gray lines indicating correlations between subclasses.

Orthogonal partial least squares discriminant analysis (OPLS‐DA) was performed, and the variable importance for projection (VIP) values were used to assess the impact and explanatory power of each lipid molecule on sample classification. The OPLS‐DA model could distinguish between the two groups (Figure 4A). The model parameter *Q* ^2^, obtained through 7‐fold cross‐validation, was 0.668, indicating the model's stability and reliability. The permutation test results showed that with decreasing permutation retention, the *R* ^2^ and *Q* ^2^ of the random model gradually decreased, indicating no overfitting in the original model and good robustness (Figure 4B and Table 3).

Based on the VIP values, lipids with VIP > 1 were considered significant contributors to the model. A total of 44 significantly different lipid molecules were identified with VIP > 1 and *p* value < 0.05 (Table S2). The top 10 upregulated lipid molecules were: Hex1Cer (d18:1/26:0), PE (16:0/16:1), PE (18:2/16:1), PE (18:0/16:1), PE (P‐18:0/16:1), Hex1Cer (d18:1/18:1), DG (16:1/18:0), PE (18:1/18:2), PC (18:1/16:1), and LPE (20:1). The top 7 downregulated lipid molecules were: TG (56:5)‐FA20:4, TG (52:3)‐FA16:0, TG (52:4)‐FA18:2, TG (52:3)‐FA18:2, DG (16:0/18:3), PE (20:5/26:6), PG (20:0/22:5) (Table 4). The bubble and heatmaps clearly illustrated the differences in lipid molecules between subclasses, with most differential lipid molecules being upregulated, while the downregulation of TG was the most significant (Figure 4C,D).

**TABLE 4:** Multivariate statistical analysis of the top 10 most significantly up‐ and top7 down‐regulated lipid molecules.

Multivariate statistical analysis	Lipid name	Class	Fold change	*p*	VIP
Upward	Hex1Cer (d18:1/26:0)	SP	5.82	0.014	1.09
	PE (16:0/16:1)	GP	2.82	0.026	1.10
	PE (18:2/16:1)	GP	2.67	0.027	1.13
	PE (18:0/16:1)	GP	2.62	0.017	1.15
	PE (P‐18:0/16:1)	GP	2.44	0.033	1.08
	Hex1Cer (d18:1/18:1)	SP	2.44	0.016	1.24
	DG (16:1/18:0)	GL	2.41	0.049	1.04
	PE (18:1/18:2)	GP	2.33	0.035	1.10
	PC (18:1/16:1)	GP	2.25	0.048	1.14
	LPE (20:1)	GP	2.18	0.040	1.12
Downward	TG (56:5)‐FA20:4	GP	0.13	0.043	1.01
	TG (52:3)‐FA16:0	GL	0.21	0.025	1.00
	TG (52:4)‐FA18:2	GL	0.22	0.038	1.04
	TG (52:3)‐FA18:2	GL	0.22	0.027	1.02
	DG (16:0/18:3)	GL	0.41	0.045	1.04
	PE (20:5/26:6)	GP	0.85	0.026	1.12
	PG (20:0/22:5)	GP	0.88	0.048	1.14

Correlation analysis of the 44 significant differential molecules produced a clustering heatmap, revealing the metabolic relationships and inter‐regulatory relationships between molecules. Lipids that showed correlated expression may be involved in the same biological process, indicating functional correlations. Additionally, positively correlated lipids might originate from the same synthetic pathway, while negatively correlated lipids might be degraded for the synthesis of other lipids, indicating synthetic transformation relationships (Figure 4E). The chord diagram shows that the inner circle links represent significantly different lipid molecules, and the outer circle arcs represent lipid subclasses. Colored lines indicate correlations within subclasses, while dark gray lines represent correlations between subclasses. PE showed a strong correlation with other differential lipid subclasses, while the downregulated TG had a weaker relationship with other subclasses, but a stronger correlation within the TG subclass (Figure 4F).

### 3.5 Chain Length and Saturation Analysis

**FIGURE 5:**
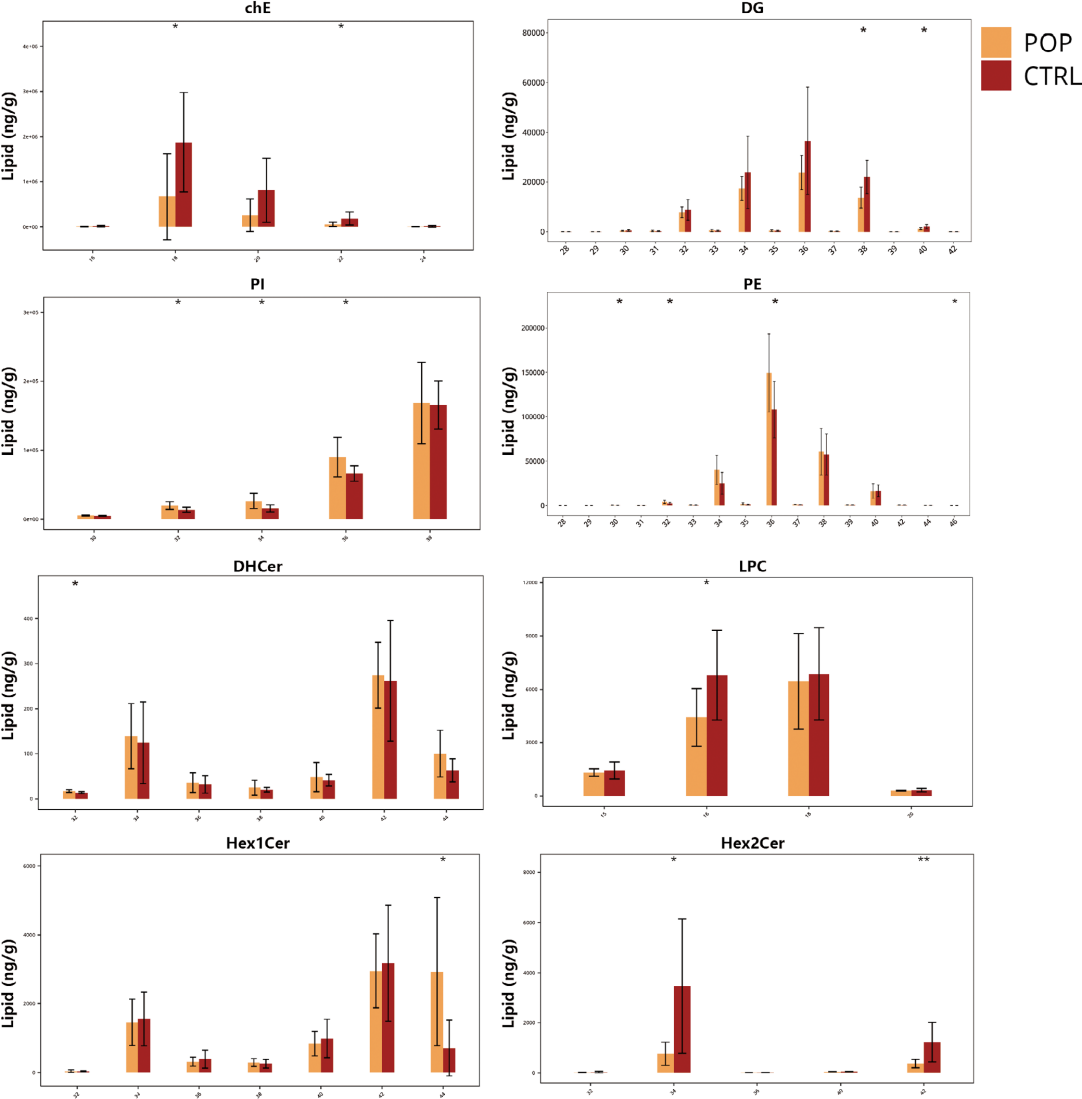
Carbon Chain Length Analysis, lipid molecules with the same chain length were summed, and differences in their abundances across different carbon chain lengths within each subclass were assessed.

Lipid molecules with identical chain lengths were aggregated and calculated the content differences for lipid molecules with different chain lengths within each subclass. Chain length may influence membrane fluidity, which can impact membrane permeability, substance transport, and membrane protein localization. Several subclasses showed changes in chain length distribution. Compared to the control group, the POP group had significantly lower contents of ChE molecules with chain lengths of 18 and 22, and DG molecules with chain lengths of 38 and 40. The content of DHCer molecules with chain length 32 significantly increased, while the content of Hex1Cer molecules with chain length 44 significantly increased. Hex2Cer molecules with chain lengths 34 and 42 significantly decreased. LPC molecules with chain length 16 significantly decreased. PE molecules with chain lengths of 30, 32, 36, and 46 significantly decreased. PI molecules with chain lengths 32, 34, and 36 significantly decreased (Figure 5).

**FIGURE 6:**
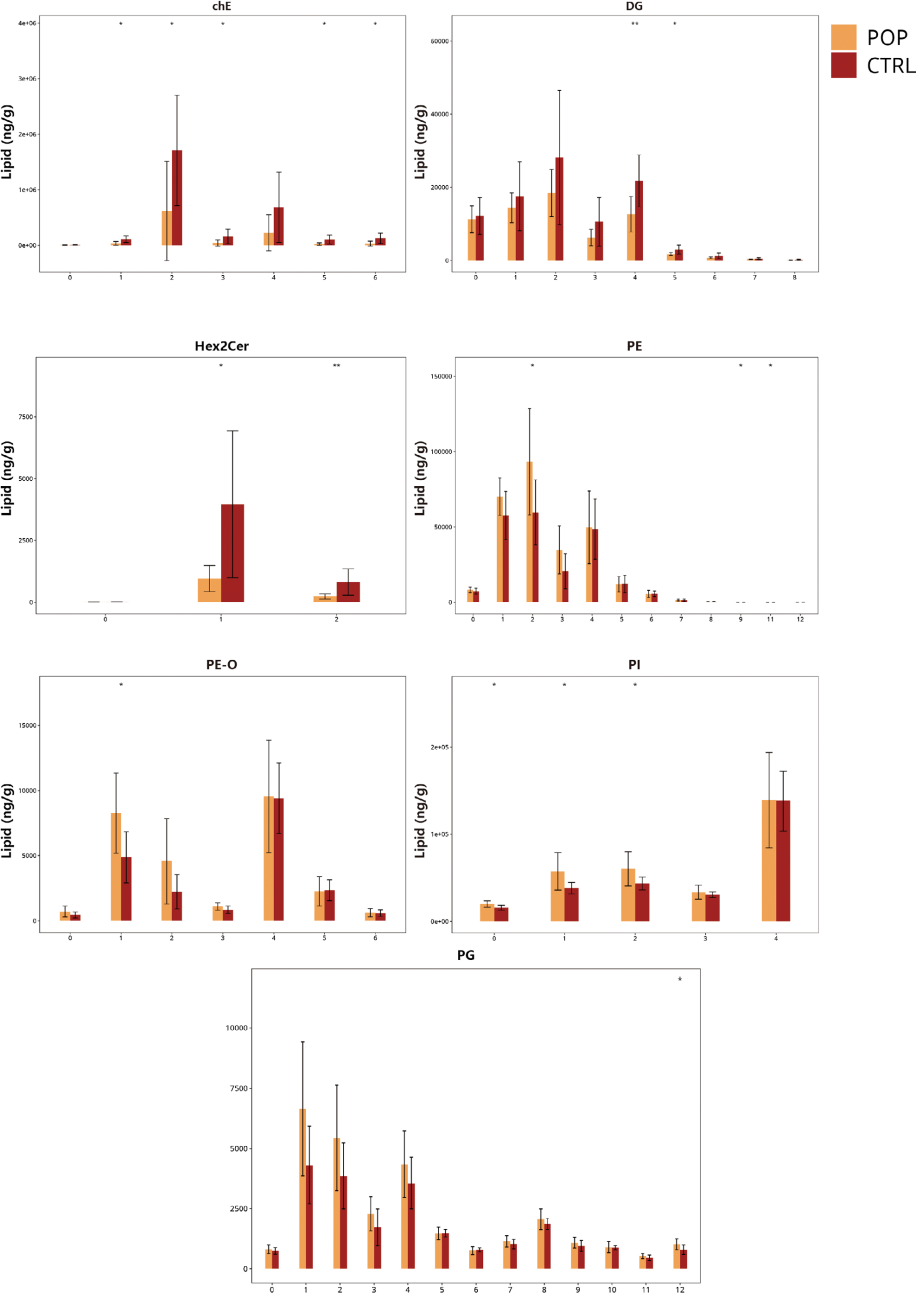
Chain Saturation Analysis, lipid molecules with the same number of double bonds were summed, and differences in their abundances across varying unsaturation levels within each subclass were assessed.

We also summed the lipid molecules with the same number of unsaturated bonds and calculated the content differences for lipid molecules with different unsaturation levels within each subclass. Compared to the control group, the POP group showed decreased unsaturation levels for ChE (with unsaturation levels 1, 2, 3, 5, and 6), DG (unsaturation levels 4 and 5), and Hex2Cer (unsaturation levels 1 and 2). PE showed increased unsaturation at levels 2, 9, and 11, PE‐O at level 1, PG at level 12, and PI at levels 0, 1, and 2 (Figure 6).

### 3.6 Enrichment Analysis

**FIGURE 7:**
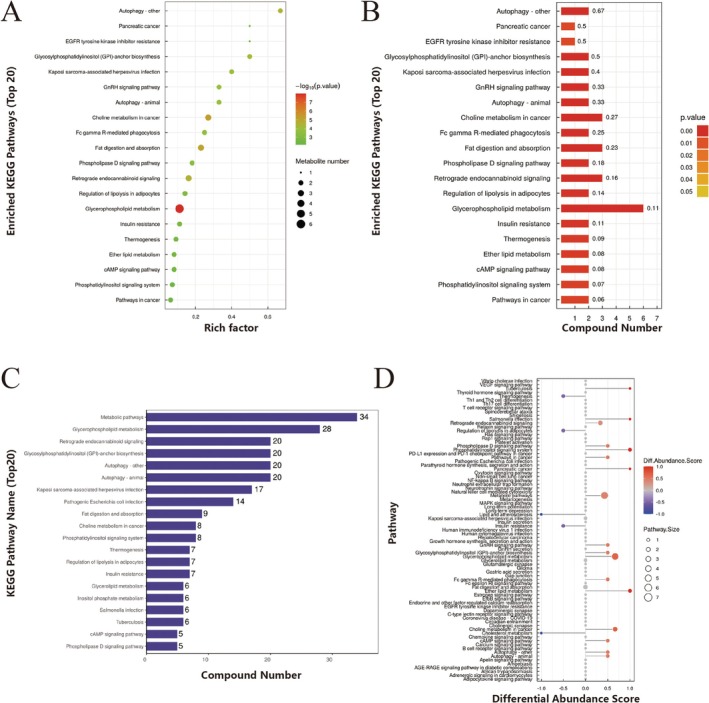
Enrichment Analysis. KEGG enrichment pathways: (A) Bubble plot and (B) Bar chart; (C) KEGG pathway annotation statistics of differential lipid molecules (Top 20); (D) Differential abundance score chart for all differential metabolic pathways.

KEGG enrichment analysis revealed that the glycerophospholipid metabolism pathway was the most significantly enriched among the differential lipid molecules (Figure 7A,B). The top 20 pathways, based on *p* value, showed the greatest number of mapped differential lipid molecules in the following five pathways: Metabolic pathways, Glycerophospholipid metabolism, Retrograde endocannabinoid signaling, Glycosylphosphatidylinositol (GPI)–anchor biosynthesis, and Autophagy—other (Figure 7C). Based on the differential abundance score (DA score) of the pathways, we found that most pathways showed an overall upregulation in expression, while a few pathways, such as Insulin resistance, were downregulated (Figure 7D).

## 4 Discussion

Using targeted lipidomics sequencing technology, our study identified a total of 1010 lipid molecules and revealed significant changes in lipid metabolism in the vaginal anterior wall support tissue of POP patients. While the overall lipid levels did not show significant changes, the notable alterations in lipid composition suggest an imbalance in lipid metabolism in POP patients. Specifically, the proportion of PA in the POP group increased dramatically (from 0.048% to 0.226%), while the proportion of TG significantly decreased (from 94.901% to 83.927%).

PA, the simplest phospholipid, comprises a glycerol backbone, two hydrophobic fatty acid side chains, and a negatively charged phosphate head. As a key metabolic intermediate and second messenger, PA is involved in various physiological processes, including mitochondrial dynamics and autophagy regulation [7]. Studies have shown that PA generates negative curvature on the outer mitochondrial membrane, stimulating mitochondrial fusion, and mediating mitochondrial fission through interactions with molecules like Drp1, thus regulating mitochondrial function [8]. Additionally, PA can directly activate the mTOR signaling pathway, participating in important functions like autophagy [9]. In POP patients, the increase in PA may reflect a cellular adaptive response to damage or stress, aiming to restore cellular function by regulating membrane stability, energy metabolism, and inflammation.

TG, the primary form of intracellular energy storage, was reduced in the POP group. This reduction may reflect a redistribution of energy metabolism or an increase in energy demand. In POP patients, the decrease in TG could be associated with increased energy consumption during tissue repair or remodeling processes. TG breakdown provides crucial substrates for mitochondrial β‐oxidation, supporting mitochondrial bioenergetic function [10]. Considering the important role of PA in mitochondrial dynamics, the reduction in TG may work in concert with the elevated PA to regulate mitochondrial function and energy metabolism, affecting the cell's ability to adapt to damage or stress.

Metabolomic analysis using the OPLS‐DA model identified significant differences in 48 lipid molecules, with PE exhibiting the most significant upregulation. PE is one of the most abundant glycerophospholipids in mammalian cell membranes, primarily synthesized through the CDP‐ethanolamine (Kennedy) pathway and the phosphatidylserine decarboxylase (PSD) pathway [11]. Studies indicate that the metabolic regulation of PE is closely associated with various physiological and pathological processes. Under chronic stress, reduced PE synthesis can lead to abnormal cell membrane structure and function, thereby promoting inflammatory responses [12].

Notably, PE is closely related to mitochondrial function. Studies show that increased PE levels within mitochondria can significantly enhance oxidative phosphorylation, while decreased PE levels activate the biogenesis of mitochondrial‐derived vesicles (MDVs) [13, 14]. This regulatory effect on mitochondrial function may be a key mechanism through which PE exerts its anti‐inflammatory effects. Specifically, PE can significantly improve palmitic acid‐induced mitochondrial dysfunction, reducing ROS and MDA levels in macrophages, thereby exerting anti‐inflammatory effects [15].

Inflammatory responses and mitochondrial dysfunction are critical in the pathogenesis of POP. Studies have shown that POP patients exhibit significantly increased levels of inflammatory cytokines (such as IL‐1, TNF‐α, IFN‐γ, etc.) in the vaginal anterior wall tissue. These cytokines may promote the pathological progression of POP by altering the collagen metabolism of the extracellular matrix (ECM) [16]. Moreover, mitochondrial dysfunction has been confirmed as an important mechanism in the pathogenesis of POP. In a vaginal injury mouse model, the pubococcygeus muscle tissue showed decreased mitochondrial membrane potential and reduced ATP generation. In human POP fibroblasts from uterosacral ligaments, mechanical damage led to ultrastructural changes in mitochondria, such as vacuolization and loss of cristae, accompanied by apoptotic body formation and compromised cell integrity [17, 18].

Based on these findings, we hypothesize that the upregulation of PE may be an adaptive response of the cells to the pathological state of POP: (1) increasing PE levels to enhance mitochondrial oxidative phosphorylation and improve cellular energy metabolism; (2) stabilizing mitochondrial structure and function by regulating mitochondrial membrane lipid composition; (3) reducing tissue damage caused by the release of inflammatory cytokines and elevated reactive oxygen species through its anti‐inflammatory properties. However, this compensatory upregulation may not be sufficient to fully reverse the pathological progression of POP, which ultimately may lead to structural and functional abnormalities of the pelvic floor support tissue. This discovery provides new research directions for a deeper understanding of the molecular mechanisms of POP and offers potential targets for the development of lipid metabolism‐based intervention strategies. Future studies can further explore strategies to optimize the lipid metabolic balance in pelvic floor tissues, such as exogenous supplementation of PE or its precursors, or regulation of key enzymes involved in PE synthesis, providing new intervention methods for the prevention and treatment of POP.

We identified several TG molecules that were significantly downregulated in POP patients. Correlation analysis showed that TG was negatively correlated with other differential lipid molecules, such as PE (except PE (20:5/26:6)), suggesting that TG may contribute to the pathological process of POP by providing precursors for the synthesis of other lipids through its catabolism. Specifically, the downregulation of TG may reflect a metabolic reprogramming in the pathological state of POP. Under chronic inflammation and oxidative stress, cells may accelerate TG breakdown to meet energy metabolism needs. Future studies could further explore the role of key TG metabolic enzymes and their metabolites in the pathological process of POP.

KEGG pathway enrichment analysis of differential lipid molecules showed the top five significantly enriched pathways: Metabolic pathways, Glycerophospholipid metabolism, Retrograde endocannabinoid signaling, Glycosylphosphatidylinositol (GPI)‐anchor biosynthesis, and Autophagy—other. These results indicate that lipid metabolism in the vaginal anterior wall of POP patients undergoes global reprogramming. Among these pathways, the GPI‐anchor biosynthesis pathway was particularly notable, suggesting that the metabolism of GPI‐anchored proteins may play a role in the pathogenesis of POP. GPI, with its unique anchoring structure, is involved in various physiological processes such as cell membrane localization, signal transduction, immune regulation, cell adhesion, and nutrient uptake, playing an important role in maintaining cell function and regulating pathological processes [19].

The length and saturation of phospholipid acyl chains determine membrane properties such as fluidity and charge distribution, thereby regulating interactions with membrane‐associated proteins. Changes in chain length and saturation directly affect membrane permeability, material transport, and membrane protein localization [20]. For example, in the skin stratum corneum of atopic dermatitis patients, ceramides with ultra‐long fatty acyl chains (C25 and above) were decreased, while ceramides with long fatty acyl chains (C24 and below) increased [21]. Changes in lipid saturation can also affect cell membrane fluidity, influencing physiological processes such as cell division, migration, and signal transduction [22]. For example, the reduction of glycerophospholipids containing palmitoyl (C16:0) can increase cell membrane fluidity, promoting the proliferation and invasiveness of liver cancer cells [23].

This study is the first to systematically analyze lipid metabolism changes in the vaginal anterior wall support tissue of POP patients using targeted lipidomics sequencing technology. Using high‐sensitivity targeted lipidomics sequencing combined with ultra‐high‐performance liquid chromatography‐mass spectrometry (UHPLC–MS/MS), we ensured the accuracy and reliability of the data. This study not only focuses on the overall changes in lipid molecules but also conducts in‐depth analyses at the subclass level, chain length, saturation, and other dimensions. This comprehensive analysis provides a theoretical basis for developing lipid metabolism‐based intervention strategies and holds potential application value. However, this study also has certain limitations. Although strict quality control and multidimensional statistical analysis ensured the reliability of the data, the limited sample size may affect the generalizability and statistical power of the results. Future research could expand the sample size to improve the reliability of the conclusions. Furthermore, lipid composition varies significantly among different cell types, and therefore the observed changes in tissue‐level lipid profiles may simply reflect differences in cellular composition between POP and control tissues. Additionally, this study still has preliminary research on the specific lipid molecular mechanisms. How changes in lipid molecules like PA affect the pathogenesis of POP through specific signaling pathways remains to be further explored.

## 5 Conclusion

This study uncovered significant alterations in lipid metabolism within the vaginal anterior wall support tissue of POP patients, including the increase in the proportion of PA/PC, decrease in TG, and upregulation of PE lipid molecules, using UHPLC–MS/MS‐based targeted lipidomics. Multilevel data analysis, encompassing lipid content, chain length, saturation, and KEGG pathways, revealed systematic and comprehensive intergroup differences. These findings highlight associations between altered lipid metabolism and POP, suggesting potential roles in cell membrane stability, mitochondrial function, and energy metabolism. Future research should further investigate the specific mechanisms of lipid metabolism regulation and develop intervention strategies based on lipid metabolism, providing new ideas for the prevention and treatment of POP.

## Author Contributions

Shufei Zhang: conceptualization, writing – original draft, visualization. Hanyue Li: writing – review and editing. Xiaoyu Tian: resources, writing – review and editing. Hongyang Xue: resources, supervision. Yong He: Investigation. Nuo Jiang: investigation. Li Hong: writing – review and editing, funding acquisition.

## Funding

This work was financially supported by the National Natural Science Foundation of China (82371639).

## Ethics Statement

This study was approved by the Clinical Research Ethics Committee of Renmin Hospital of Wuhan University (WDRY2025‐K018). All procedures were performed in accordance with the ethical standards of the Declaration of Helsinki.

## Consent

Written informed consent was obtained from all participants and/or their legal guardians before sample collection.

## Conflicts of Interest

The authors declare no conflicts of interest.

## Supporting information


**Figure S1:** Overall Differential Analysis and PCA Results. (A) Overall lipid content differential statistics chart; (B) PCA score plot; (C) PLS‐DA score plot; (D) PLS‐DA permutation test plot.

**Table S1:** Univariate statistical analysis of the up‐ and down‐regulated lipid molecules.
**Table S2:** Multivariate Statistical analysis of the up‐ and down‐regulated lipid molecules.

## Data Availability

The reported data is also available with the corresponding author and can be accessed upon submitting a reasonable request.
