# Supplementary material for: Altered Lipid Metabolism in the Vaginal Wall of Women With Pelvic Organ Prolapse: A Targeted Lipidomics Study
Source: FASEB J. 2025 Nov 28;39(23):e71271. doi: 10.1096/fj.202502840R (PMC12662263; doi:10.1096/fj.202502840R)
Supplement: Supplementary file 1 — Figure S1: Overall Differential Analysis and PCA Results. (A) Overall lipid content differential statistics chart; (B) PCA score plot; (C) PLS‐DA score plot; (D) PLS‐DA permutation test plot. [file FSB2-39-e71271-s002.docx]

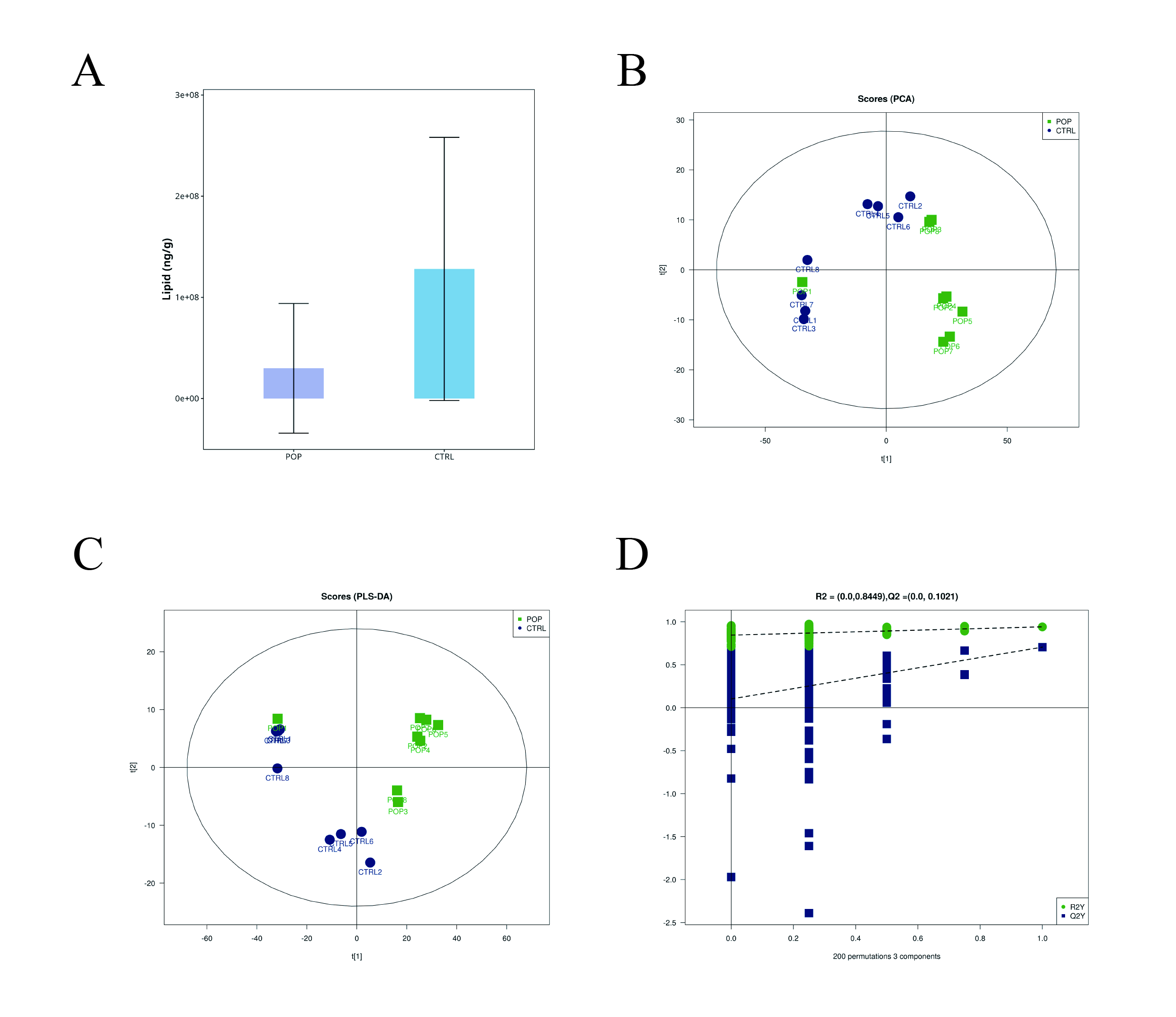


**Sup Figure 1:** Overall Differential Analysis and PCA Results. (A) Overall lipid content differential statistics chart; (B) PCA score plot; (C) PLS-DA score plot; (D) PLS-DA permutation test plot.
