# Supplementary material for: Altered Lipid Metabolism in the Vaginal Wall of Women With Pelvic Organ Prolapse: A Targeted Lipidomics Study
Source: FASEB J. 2025 Nov 28;39(23):e71271. doi: 10.1096/fj.202502840R (PMC12662263; doi:10.1096/fj.202502840R)
Supplement: Supplementary file 2 — Table S1: Univariate statistical analysis of the up‐ and down‐regulated lipid molecules. Table S2: Multivariate Statistical analysis of the up‐ and down‐regulated lipid molecules. [file FSB2-39-e71271-s001.docx]

Sup Table 1. Univariate statistical analysis of the up- and down-regulated lipid molecules

| Upward | Downward |
| --- | --- |
| Hex1Cer(d18:1/26:0) | PG(20:0/22:5) |
| PI(18:0/18:1) | PE(20:5/26:6) |
| PI(16:0/20:3) | Hex1Cer(d18:0/24:1) |
| PI(16:0/18:2) | LPC(16:0) |
| PI(16:0/18:1) | DG(16:1/20:4) |
| PI(16:0/16:0) | PE(P-20:1/18:1) |
| PI(15:0/17:0) | PE(P-20:1/20:3) |
| PG(22:6/22:6) | DG(18:0/20:4) |
| PE(P-18:0/16:1) | DG(18:1/20:4) |
| PE(P-16:0/18:3) | DG(18:0/22:5) |
| PE(O-18:0/18:1) | DG(18:1/20:0) |
| PE(O-16:0/20:1) | DG(16:0/18:3) |
| PE(O-16:0/18:0) | ChE(18:2) |
| PE(20:5/22:4) | ChE(18:1) |
| PE(18:2/20:1) | Hex2Cer(d18:1/24:0) |
| PE(18:2/20:0) | Hex2Cer(d18:1/24:1) |
| PE(18:2/18:3) | ChE(20:3) |
| PE(18:2/18:2) | ChE(22:6) |
| PE(18:2/16:1) | TG(52:2)-FA18:1 |
| PE(18:1/20:1) | TG(54:3)-FA18:1 |
| PE(18:1/18:2) | Hex2Cer(d18:1/16:0) |
| PE(18:1/18:1) | TG(52:3)-FA18:1 |
| PE(18:1/16:1) | TG(52:2)-FA16:0 |
| PE(18:0/16:1) | TG(54:4)-FA18:1 |
| PE(16:0/16:1) | TG(54:4)-FA18:2 |
| PE(16:0/14:0) | TG(52:3)-FA18:2 |
| PC(18:1/16:1) | TG(52:4)-FA18:2 |
| PC(15:0/18:0) | TG(52:3)-FA16:0 |
| PC(15:0/15:0) | ChE(20:5) |
| PA(18:1/20:1) | TG(56:5)-FA20:4 |
| PA(16:0/16:1) | TG(58:9)-FA20:4 |
| LPE(20:1) | / |
| LPE(16:1) | / |
| Hex1Cer(d18:1/18:1) | / |
| Hex1Cer(d18:0/26:0) | / |
| DHCer(d18:0/14:0) | / |
| DG(18:0/20:2) | / |
| DG(16:1/18:0) | / |

Sup Table 2. Multivariate Statistical statistical analysis of the up- and down-regulated lipid molecules

| Lipid Name | Lipid Modulation | Lipid Name | Lipid Modulation |
| --- | --- | --- | --- |
| Hex1Cer(d18:1/26:0) | Upward | PE(18:2/20:0) | Upward |
| PE(16:0/16:1) | Upward | Hex1Cer(d18:0/26:0) | Upward |
| PE(18:2/16:1) | Upward | PE(O-18:0/18:1) | Upward |
| PE(18:0/16:1) | Upward | PI(18:0/18:1) | Upward |
| PE(P-18:0/16:1) | Upward | PE(O-16:0/18:0) | Upward |
| Hex1Cer(d18:1/18:1) | Upward | PE(18:2/18:3) | Upward |
| DG(16:1/18:0) | Upward | PE(18:1/20:1) | Upward |
| PE(18:1/18:2) | Upward | PI(15:0/17:0) | Upward |
| PC(18:1/16:1) | Upward | PI(16:0/16:0) | Upward |
| LPE(20:1) | Upward | PC(15:0/15:0) | Upward |
| PE(18:2/18:2) | Upward | PC(15:0/18:0) | Upward |
| PE(18:1/16:1) | Upward | PG(22:6/22:6) | Upward |
| PE(18:1/18:1) | Upward | PI(16:0/20:3) | Upward |
| PE(P-16:0/18:3) | Upward | DHCer(d18:0/14:0) | Upward |
| PE(18:2/20:1) | Upward | PE(20:5/22:4) | Upward |
| PI(16:0/18:2) | Upward | PG(20:0/22:5) | Downward |
| PI(16:0/18:1) | Upward | PE(20:5/26:6) | Downward |
| PE(O-16:0/20:1) | Upward | DG(16:0/18:3) | Downward |
| DG(18:0/20:2) | Upward | TG(52:3)-FA18:2 | Downward |
| PE(16:0/14:0) | Upward | TG(52:4)-FA18:2 | Downward |
| PA(16:0/16:1) | Upward | TG(52:3)-FA16:0 | Downward |
| PA(18:1/20:1) | Upward | TG(56:5)-FA20:4 | Downward |
